# Supplementary material for: Structural and Functional Support by Left Atrial Appendage Transplant to the Left Ventricle after a Myocardial Infarction
Source: Int J Mol Sci. 2022 Apr 22;23(9):4661. doi: 10.3390/ijms23094661 (PMC9104858; doi:10.3390/ijms23094661)
Supplement: Supplementary file 1 [file ijms-23-04661-s001.zip › S1 Figure - Supllementary Figure S1_Leinonen.pdf]

MYOCARDIAL INFARCTION (MI) & LEFT ATRIAL APPENDAGE (LAA) TRANSPLANTATION SURGERY

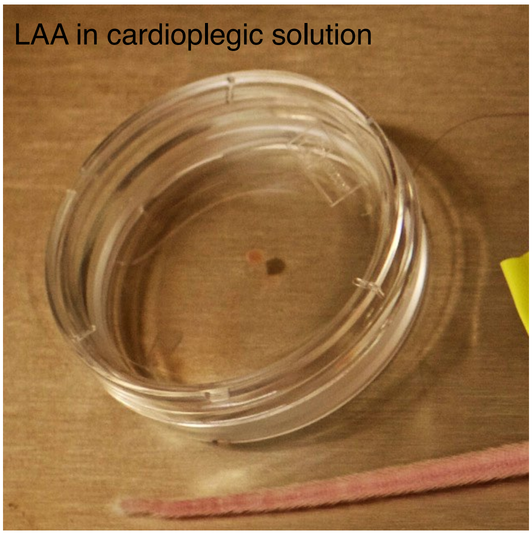

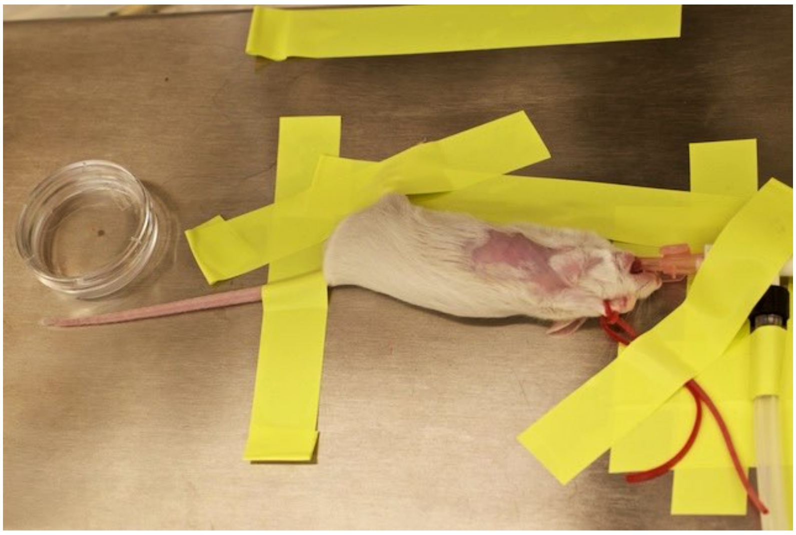

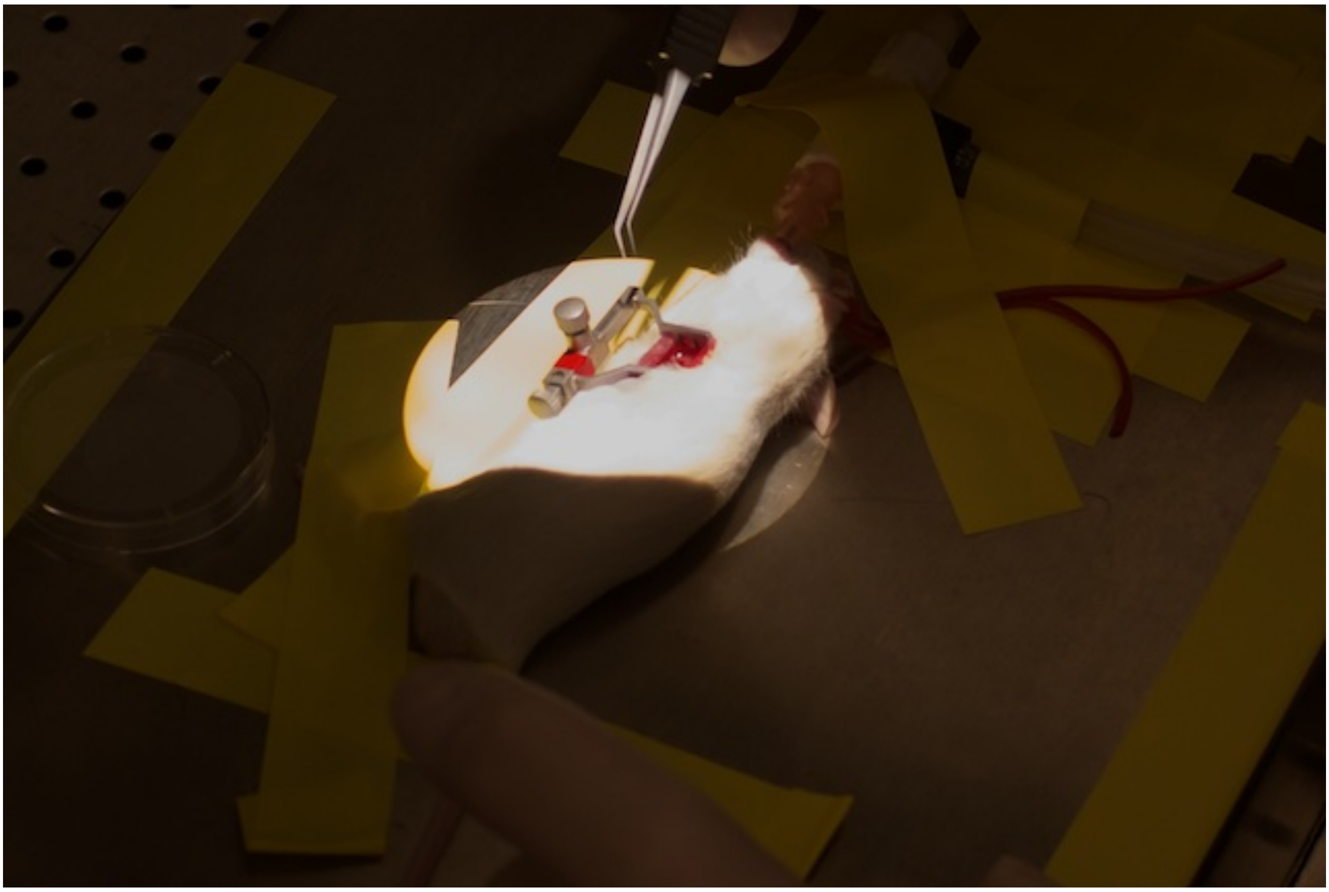

MI

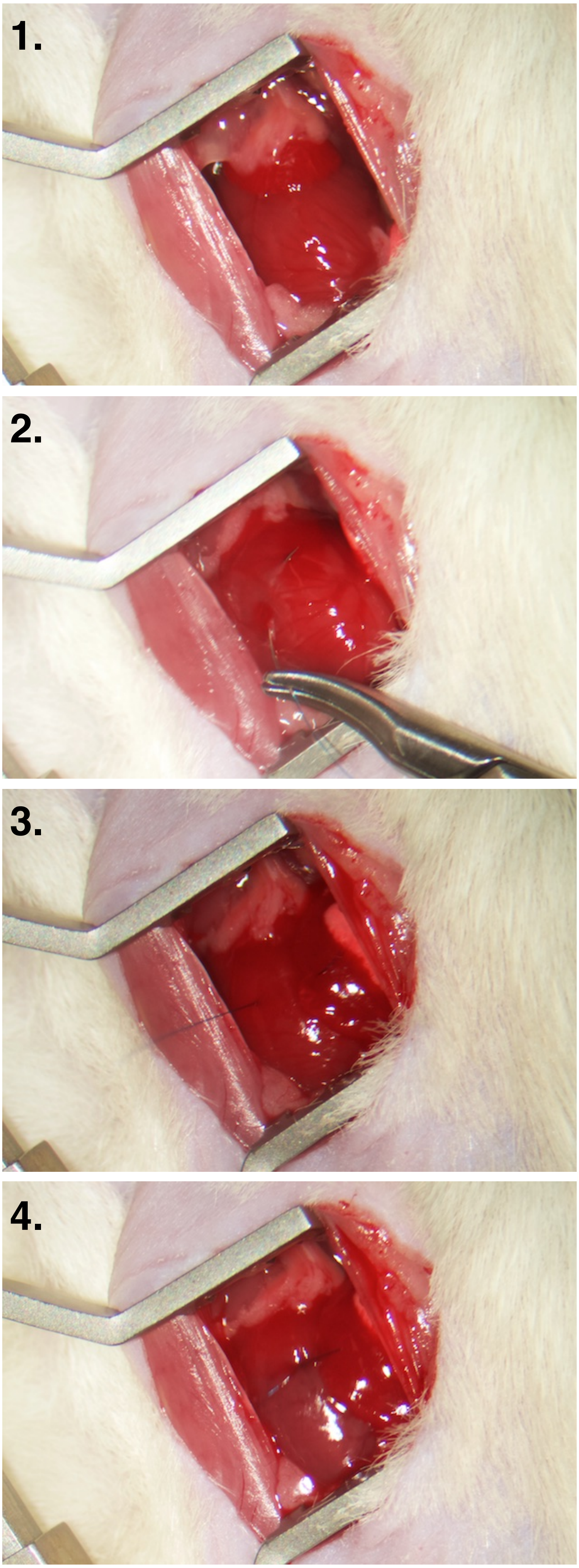

LAA TRANSPLANT

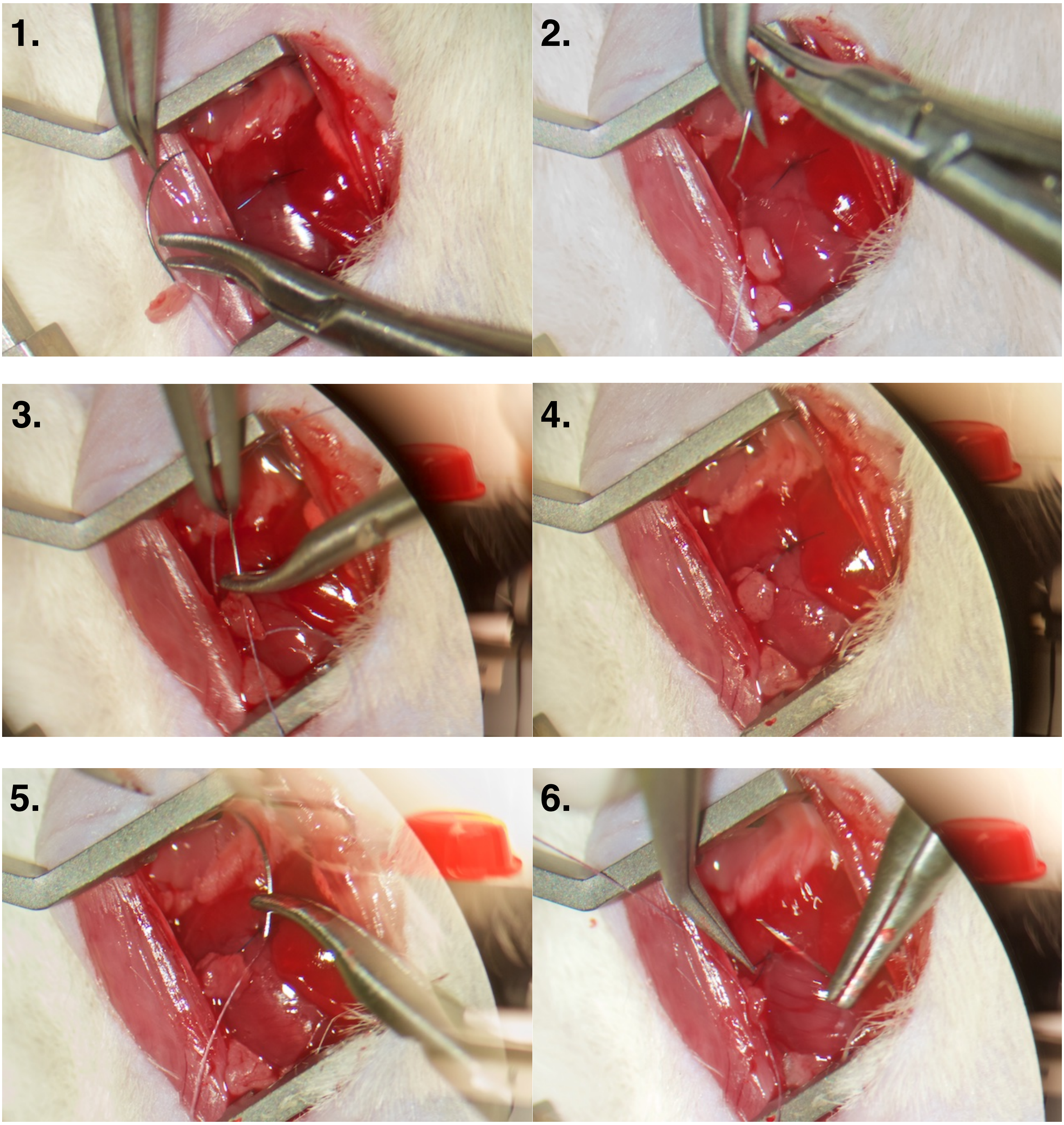

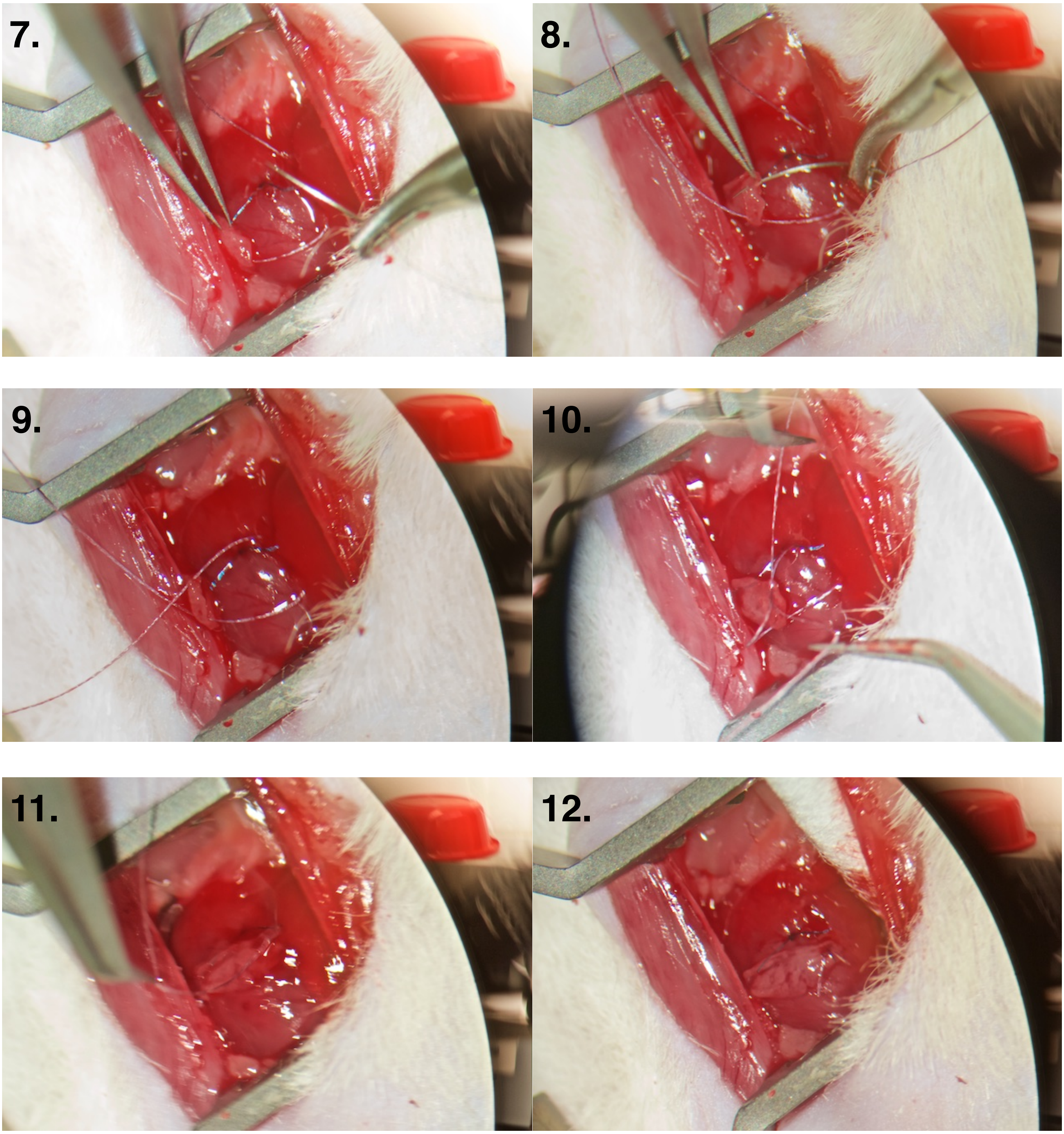
7.
8.
9.
10.
11.
12.
